# The Impact of Diabetes Mellitus and Admission Hyperglycemia on Clinical Outcomes after Recanalization Therapies for Acute Ischemic Stroke: STAY ALIVE National Prospective Registry

**DOI:** 10.3390/life12050632

**Published:** 2022-04-25

**Authors:** Peter Janos Kalmar, Gabor Tarkanyi, Zsofia Nozomi Karadi, Laszlo Szapary, Edit Bosnyak

**Affiliations:** Department of Neurology, Medical School, University of Pecs, 7624 Pecs, Hungary; kalmar.peter@pte.hu (P.J.K.); tarkanyi.gabor@pte.hu (G.T.); karadi.zsofia@pte.hu (Z.N.K.); bosnyak.edit@pte.hu (E.B.)

**Keywords:** stroke, thrombectomy, hyperglycemia, diabetes mellitus, thrombolysis

## Abstract

It was previously reported that diabetes mellitus (DM) and admission hyperglycemia (aHG) were associated with poor clinical outcomes in patients with acute ischemic stroke (AIS) who were treated with intravenous thrombolysis (IVT) or mechanical thrombectomy (MT). Our study aimed to assess the prognostic effect of DM and aHG (≥7.8 mmol/L) on clinical outcomes in patients treated with recanalization therapies (IVT and MT). Our multicentric study was based on data from the prospective STAY ALIVE stroke registry between November 2017 and January 2020. We compared the demographic data, clinical parameters and time metrics between recanalized DM and non-DM groups, and we analyzed the impact of DM and aHG on 90-day functional outcome, 90-day mortality, symptomatic intracranial hemorrhage (sICH), and successful recanalization. Statistical analyses were also performed in two subgroups: (1) patients treated with IVT alone and (2) patients treated with MT. Altogether, we included 695 patients from the three participating stroke centers in Hungary. Regarding the overall population, patients with diabetes were older (72 vs. 67 years, *p* < 0.001) and comorbidities were more frequent. There were significant differences in the 90-day good functional outcome (48.9% vs. 66.7%, *p* < 0.001), 90-day mortality (21.9% vs. 11.6%, *p* < 0.001) and the rate of symptomatic intracranial hemorrhaging (sICH) (7.8% vs. 2.2%, *p* < 0.001) between the groups. Diabetes and aHG were independently associated with a poor clinical outcome (OR 2.02, 95% CI 1.31–3.11, *p* = 0.001; OR 2.09, 95% CI 1.39–3.14, *p* < 0.001) and mortality at 3 months (OR 2.45, 95% CI 1.35–4.47, *p* = 0.003; OR 2.42, 95% CI 1.37–4.28, *p* = 0.002) and sICH (OR 4.32, 95% CI 1.54–12.09, *p* = 0.005; OR 4.61, 95% CI 1.58–13.39, *p* = 0.005) in the overall population. However, the presence of DM and aHG was not correlated with successful reperfusion (OR 0.39, 95% CI 0.09–1.67, *p* = 0.205; OR 0.42, 95% CI 0.09–1.97, *p* = 0.274) after MT. Our study revealed that diabetes and hyperglycemia on admission were correlated with poor clinical outcomes at 3 months in patients with acute stroke regardless of the recanalization method. In addition, the variables were also associated with sICH after recanalization therapies. However, successful recanalization was not associated with DM and aHG in patients who underwent MT.

## 1. Introduction

Acute and chronic hyperglycemia are important risk factors for acute ischemic stroke (AIS). Patients with diabetes mellitus (DM) have more than two times higher risk of AIS compared to non-diabetics [1]. In addition, stroke outcomes are worse in diabetic patients, as diabetes has adverse effects on the vasculature including endothelial dysfunction, inflammation, and prothrombotic state [2,3,4]. Furthermore, diabetes also leads to a decrease in the size and the number of branches of lentriculostriate arteries that potentially affect the local cerebral circulation leading to greater ischemic deficits and delayed recovery after stroke [5]. 

On the other hand, admission hyperglycemia (aHG) occurs in 30–40% of patients with AIS, even if they do not have a history of diabetes [1]. Potential detrimental mechanisms of aHG include disruption of the blood–brain barrier, lactic acid accumulation in the ischemic area generating more extensive infarction, and the inhibition of plasma fibrinolysis [6,7]. In the National Institute of Neurological Disorders and Stroke recombinant tissue plasminogen activator (NINDS rt-PA) trial, patients with increased baseline glucose values were associated with longer periods of hospitalization, increased cost, and death [8]. Previous studies showed that aHG was correlated with lower reperfusion rates in patients with AIS after intravenous thrombolysis (IVT) [9,10,11]. However, other research reported conflicting results, as they found that the presence of aHG was not correlated with significantly worse outcomes in patients who underwent IVT [12]. 

Furthermore, evidence is limited for stroke patients with acute stroke who were treated with intra-arterial mechanical thrombectomy (MT). Only a few previous studies found that aHG may increase the risk of poor functional outcomes after MT, especially in patients with incomplete reperfusion [13,14]. The post hoc analysis of previous randomized controlled trials showed inconsistent results regarding the potential negative effect of elevated serum glucose values on clinical outcomes. In the analysis of the Solitaire Flow Restoration with the Intention for Thrombectomy (SWIFT) trial, hyperglycemia was correlated with a 90-day poor functional outcome [13]. However, in another research work based on data from the MR CLEAN trial, there was no significant relationship between elevated plasma glucose concentration and clinical outcomes in patients with AIS who were treated with MT [15]. 

Our study aimed to assess the prognostic impact of DM and aHG on functional and clinical outcomes of AIS patients who were treated with recanalization therapies. Moreover, we sought to investigate the potentially detrimental effects of these risk factors on outcomes with different recanalization techniques.

## 2. Materials and Methods

### 2.1. Study Population

Data of patients with AIS who received recanalization treatment (IVT-only or MT) between November 2017 and January 2020 were prospectively collected from our multicentric stroke registry (STAY ALIVE Acute Stroke Registry) and were retrospectively analyzed. Three comprehensive stroke institutes in Hungary participated in the present study. The research protocol was approved by the Hungarian ethics committee. All involved patients gave written consent to participate in our study, following the Good Clinical Practice (GCP) guidelines.

### 2.2. Diagnosis

Patients were admitted mainly to the emergency department (ED) of the stroke centers or the hospitals of surrounding cities and were transferred to comprehensive vascular centers. Patients were examined immediately after admission based on the standard examination protocol (including medical history and recording of stroke risk factors, blood test, electrocardiography) and the baseline National Institutes of Health Stroke Scale (NIHSS) which was assessed by a dedicated stroke neurologist. Baseline plasma glucose levels were measured before the recanalization therapy at the ED. The aHG was defined as the first serum glucose value ≥7.8 mmol/L as per the criteria of the American Diabetes Association [16]. DM was defined based on a history of diabetes or the use of insulin and/or an oral antidiabetic agent.

Every patient underwent cranial CT based on the stroke imaging protocol of the centers to evaluate the intra- and extracranial vessel status and to exclude intracranial bleeding events. The standard CT imaging protocol included a non-contrast CT scan, CT angiography, and CT perfusion. All baseline parameters were recorded in our stroke registry. We recorded and investigated the following parameters: (a) demographic data; (b) vascular risk factors; (c) baseline, 24 h, and 72 h neurological status based on NIHSS score; (d) imaging data (Alberta Stroke Program Early CT Score [ASPECTS]; multiphase CT-angiography [mCTA] collateral score); (e) time metrics, details of the recanalization treatment and complications.

### 2.3. Treatment

All investigated patients received recanalization therapy. In every eligible patient, standard IVT therapy was applied (0.9 mg/kg) within 4.5 h after symptom onset (according to the conventional criteria of IVT application). Patients meeting the following criteria underwent mechanical thrombectomy: (1) age over 18 years; (2) acute stroke symptoms with NIHSS score ≥ 4 or isolated aphasia or hemianopia; (3) symptoms persisted for up to 12 h; (4) large vessel occlusion (LVO) detected by CT angiography and/or peri-interventional angiograms. Endovascular therapy involved arterial catheterization to the LVO followed by MT. A direct aspiration catheter was primarily used for the MT or applied in combination with a stent-retriever. The exact endovascular strategy was left to the decision of the neurointerventional specialist. The final mTICI scoring of 2b, 2c, or 3 (modified thrombolysis in cerebral infarction) was defined as successful recanalization [17].

A control CT scan was performed after 24 h (or if there was any sign of clinical deterioration) from stroke onset to assess the postprocedural intracranial status. The intracranial bleeding events were classified according to the European Cooperative Acute Stroke Study (ECASS II) classification [18]. Symptomatic intracranial hemorrhage (sICH) was defined as parenchymal hematoma (PH1 and PH2) on the control imaging and associated with a 4-point increase in the NIHSS.

### 2.4. Outcomes

The primary outcome was considered a good (independent) functional outcome (modified Rankin Scale (mRS) score 0–2) at 90 days. Other analyzed outcomes included poor functional outcome (mRS 3–6) and all-cause mortality at 90 days, successful arterial recanalization (TICI ≥ 2b), and symptomatic intracranial hemorrhage (sICH).

### 2.5. Statistical Analysis

For the statistical data analysis, SPSS (version 26.0, IBM, New York, NY, USA) was applied. Normality was assessed with the Kolmogorov–Smirnov test. Quantitative data were expressed as the median and interquartile range (IQR) or the mean ± standard deviation (SD). Categorical variables were analyzed using chi-square or the Fisher’s exact test. For the comparison of the continuous variables, Student *t*-tests or Mann–Whitney U tests were used. Multivariable binary logistic regression analysis was performed to analyze the relationship between diabetes, aHG, and the investigated outcomes. For the adjustment of potential confounders, variables with a *p*-value < 0.2 in the univariate analysis were entered into the multivariable logistic regression model. Statistical analyses were also performed in the 2 subgroups: (1) patients treated with MT and (2) patients treated with IVT-only. Statistical significance was considered as *p* ≤ 0.05.

## 3. Results

### 3.1. Results of the Overall Population

Altogether we enrolled 695 (45.0% women) patients with ischemic stroke who received recanalization therapies (IVT-only or MT) between November 2017 and January 2020 at the three participating stroke centers. Eighty-one percent of all patients received IVT-only and MT was performed in 130 (19%) cases. The clinical characteristics of enrolled patients are summarized in Table 1. 

The median age was 69 years (IQR: 60–77). Diabetes was present in 182 (26.2%) patients. Diabetic patients were older (72 vs. 67 years, *p* <0.001) and had a higher body mass index (BMI) (28.1 vs. 25.8 kg/m^2^, *p* < 0.001). The prevalence of several comorbidities was significantly higher in the DM group, such as hypertension (92.7% vs. 74.5%, *p* < 0.001), atrial fibrillation (23.0% vs. 15.6%, *p* = 0.027) and coronary artery disease (41.0 vs. 18.2%, *p* < 0.001). Regarding the admission parameters, the median NIHSS score on admission was 7 points (IQR 5–11), with a median ASPECTS of 10 points (IQR 9–10) and a median mCTA score of 5 points (IQR 4–5). Mean admission serum glucose was 6.6 mmol/L (IQR 5.8–8.3). Moreover, the rate of aHG (60.6% vs. 20.0%, *p* < 0.001), the admission blood glucose values (8.7 vs. 6.4 mmol/L, *p* < 0.001) and creatinine values (91 vs. 81 mg/dL, *p* < 0.001) were higher in the diabetic population. After the 90-day follow-up, the rate of favorable outcomes was 62.1% and the mortality rate was 14.3%. Poor functional outcome at 90 days was more frequent in DM patients (51.1% vs. 33.3%, *p* < 0.001), who also had significantly higher mortality rates (21.9% vs. 11.6%, *p* < 0.001). In addition, sICH was detected in 25 (3.6%) patients, and there was a higher incidence of intracranial bleeding events (7.8% vs. 2.2%, *p* = 0.001) in the DM group.

After adjusting for potential confounders, DM and aHG were independently associated with 90-day poor functional outcome (OR 2.02, 95% CI 1.31–3.11, *p* = 0.001; OR 2.09, 95% CI 1.39–3.14, *p* < 0.001), 90-day mortality (OR 2.45, 95% CI 1.35–4.47, *p* = 0.003; OR 2.42, 95% CI 1.37–4.28, *p* = 0.002) and sICH (OR 4.32, 95% CI 1.54–12.09, *p* = 0.005; OR 4.61, 95% CI 1.58–13.39, *p* = 0.005). The relation between DM, aHG and clinical outcomes is summarized in Table 2. 

Since the size of occluded intracranial vessels, the severity of the stroke, and the method of recanalization also differed between the patients, we performed subgroup analysis to compare homogenous patient groups.

### 3.2. Results of Subgroup Treated with MT

A total of 130 patients (53.1% female) with LVO were treated with MT, and 33 of them (25.4%) had been previously diagnosed with diabetes. The characteristics of the MT subgroup are summarized in Table 3. The mean age was 68 years (IQR 60–79). The clinical and laboratory parameters on admission did not differ significantly between the groups. However, patients with diabetes had higher baseline serum glucose levels (7.9 vs. 6.6 mmol/L, *p* = 0.001) and aHG was more common (59.3% vs. 25.3%, *p* = 0.001). In addition, the rate of eligible patients for combined therapy with IVT was lower (21.2% vs. 47.4%, *p* = 0.008) in the diabetic group. Regarding the details of the intervention, the median symptom onset to arterial puncture time was 230 min (IQR 182–300). The time required from the symptom onset to revascularization was moderately longer in the DM group (305 vs. 257 min, *p* = 0.192); however, the difference was not significant. Furthermore, there was no significant difference, in the rate of successful recanalization (81.8% vs. 89.6%, *p* = 0.243), and intraprocedural complications (10.3% vs. 9.5%, *p* = 0.898), although the intracranial bleeding events were more common (18.2% vs. 6.2%, *p* = 0.040) in patients with diabetes. The rate of good functional outcome at 90 months was significantly higher (43.3% vs. 24.2%, *p* = 0.046) in the non-DM patient group with a significantly lower 90-day mortality rate (21.6% vs. 39.3, *p* = 0.037). 

The correlation between DM, aHG, and clinical outcomes at 90 days is presented in Table 4. There was unadjusted relative risk between DM, aHG, and 90-day poor functional outcome, 90-day mortality, and sICH. In multivariable analysis, DM and aHG were independently correlated with mortality at 90 days (OR 3.72, 95% CI 1.04–13.34, *p* = 0.044; OR 3.76, 95% CI 1.11–12.76, *p* = 0.034) and hemorrhagic transformations (OR 12.45, 95% CI 1.73–89.60, *p* = 0.012; OR 7.36, 95% CI 1.26–44.18, *p* = 0.029). In addition, aHG was independently associated with poor functional outcomes at 90 days (OR 6.99, 95% CI 1.98–24.72, *p* = 0.003). The presence of DM and aHG was not associated with successful reperfusion (OR 0.39, 95% CI 0.09–1.67, *p* = 0.205; OR 0.42, 95% CI 0.09–1.97, *p* = 0.274) after MT.

### 3.3. Results of Subgroup Treated with IVT-Only

Patients were predominantly treated with IVT alone (81.3%). Therefore, the patients’ characteristics were very similar compared to the results of the overall population. The details of the IVT-only subgroup are presented in Table S1 in the Supplementary Material. DM and aHG were independently associated with the analyzed clinical outcomes in multivariable analysis (Table S2). 

## 4. Discussion

In this multicentric study, DM and aHG were associated with 90-day poor functional outcome, 90-day mortality, and increased risk of sICH in recanalized stroke patients regardless of the recanalization method. After adjusting for covariates, the relationship remained independent between clinical outcomes and risk factors in the overall population. Our findings are consistent with previous studies that identified diabetes and elevated serum glucose on admission as important predictors of unfavorable outcomes in AIS patients treated with recanalization therapies [19,20]. However, these studies were predominantly single-center and investigated the impact of diabetes and aHG separately on outcomes. In contrast, we assessed the relationship between DM, aHG, and clinical outcomes in parallel, and thereby we sought to reduce the heterogeneity of demographic and baseline characteristics in the patient groups. 

Multiple animal experiments have reported that elevated serum glucose is associated with a more extensive infarct area compared to normal blood glucose levels [21,22]. In the background of the pathological mechanism of hyperglycemia, the acceleration of infarct progression, mainly in the ischemic penumbra, has been described [23,24]. Previous research revealed potential mechanisms related to more severe neuronal damage in acute ischemic tissues including the accumulation of extracellular glutamate, intracellular acidosis, increased blood–brain barrier disruption, the development of cerebral edema, and impaired plasma fibrinolysis [6,7]. 

Furthermore, the effect of the investigated variables on clinical outcomes is unclear in patients who were treated with MT due to conflicting results in post hoc analyses of previous randomized trials. The analysis of the MR CLEAN trial found no correlation between admission serum glucose level and clinical outcomes after 90 days [15]. In contrast, the research based on data from the SWIFT trial identified an independent association between aHG and worse outcomes at 90 days, although the results only reach statistical significance in patients with incomplete reperfusion [13]. Moreover, the correlation between diabetes and clinical outcomes was not investigated in the aforementioned studies. 

In a multicentric Chinese study with a similar MT case number, an independent association was found between aHG and sICH and poor functional independence at 90 days in patients who underwent solitaire stent thrombectomy. However, they applied only a Solitaire stent-retriever device, and aHG was not identified as an independent predictor of mortality at 3 months [25]. In contrast, in our study, the aspiration technique was used predominantly, and we found a significant relationship between aHG and 90-day mortality in multivariable analysis. 

Previously, observational studies demonstrated the independent correlation between elevated serum glucose levels, diabetes, and lower recanalization rates in patients treated with IVT alone [9,10,11]. Jiang et al. found precipitating factors leading to impaired fibrinolytic response after tPA treatment including diabetic vasculopathy, elevated plasminogen activator inhibitor (PAI-1) value, glycation of tPA receptor annexin A2, and increased density of fibrin clot [26]. However, the post hoc analysis of the SWIFT trial found that aHG was not correlated with the rate of recanalization in thrombectomized patients. Our results are in line with these data because there was no significant difference in the reperfusion status between the groups. In addition, successful recanalization was not correlated with DM or aHG in patients who underwent MT. These results suggest that MT patients with the investigated risk factors could overcome the potentially reduced fibrinolytic capacity. However, in patients treated with MT, the impact of diabetes and aHG on clinical outcomes is likely to include greater reperfusion injury and collateral circulation damage. 

In our present study, symptomatic intracerebral hemorrhagic transformation occurred 25 times. The frequency of sICH was significantly higher in diabetic patients in all analyzed groups. Furthermore, sICH was also independently associated with DM and hyperglycemia, regardless of whether the treatment was IVT alone or MT. Previously reported potential pathological factors include vascular oxidative stress, neuroinflammation, extracellular proteolysis dysfunction, and blood–brain barrier disruption. These may be responsible for the higher possibility of sICH after tPA treatment [26]. Moreover, our results are consistent with clinical studies reporting that aHG was correlated with an elevated sICH rate in AIS patients who underwent MT [25,27]. It may be necessary to investigate other factors and biomarkers to understand the exact underlying mechanism [28]. 

If there is a causal link between hyperglycemia and poor outcome after AIS, lowering serum glucose levels may improve the clinical outcome. However, previous trials failed to confirm the positive association between post-stroke glucose-lowering therapy and favorable clinical outcomes. In a Cochrane review that summarized the results of 11 randomized controlled trials including 1583 patients, the authors found that intensive glucose control with intravenous insulin did not prove to be beneficial regarding functional outcomes, mortality, or improvement in final neurological deficits, but the number of hypoglycemic episodes was significantly increased [29]. However, these trials predominantly included patients treated with IVT alone (87%), the authors did not perform subgroup analyses according to different recanalization therapies, and the recanalization status of the enrolled patients was not investigated. 

The main strength of our study is that it relied on multicentric prospective data with a large patient number. In addition, DM and aHG were assessed in parallel with subgroup analysis based on the recanalization method. The applied aspiration MT technique should also be highlighted as in the previous studies primarily MT with stent-retriever was investigated. However, the present study has some potential limitations. First, this is a post hoc analysis of our prospective stroke registry. Second, glycated hemoglobin A1C (HbA1C) was not assessed. Therefore, some patients with undiagnosed diabetes may have been missed. Moreover, we found a statistically significant relationship between diabetes and poor functional outcome in the MT subgroup, but the correlation did not remain independent in the multivariable analysis due to the small sample size, which calls for larger studies to confirm our observations.

## 5. Conclusions

Our study revealed that DM and hyperglycemia on admission were correlated with poor clinical outcomes at 90 days in acute stroke patients regardless of the recanalization method. In addition, the variables were also associated with sICH after recanalization therapies. However, successful arterial recanalization was not associated with DM and aHG in patients who underwent MT.

## Figures and Tables

**Table 1:** Baseline and evaluated clinical characteristics of the overall population.

	Overall Population (*n* = 695)	DM Present (*n* = 182)	DM Absent (*n* = 513)	*p*-Value
Age, years, median (IQR)	69 (60–77)	72 (64–78)	67 (59–77)	<0.001
Gender, female, % (*n*)	45.0 (313)	46.7 (85)	44.4 (228)	0.599
Hypertension, % (*n*)	79.2 (548)	92.7 (166)	74.5 (382)	<0.001
Hyperlipidemia, % (*n*)	62.1 (394)	69.1 (114)	59.7 (280)	0.032
Atrial fibrillation, % (*n*)	17.5 (119)	23.0 (40)	15.6 (79)	0.027
Coronary artery disease, % (*n*)	23.9 (157)	41.0 (68)	18.2 (89)	<0.001
Previous stroke/TIA, % (*n*)	22.0 (148)	29.2 (49)	19.6 (99)	0.009
Antiplatelet therapy on admission, % (*n*)	37.4 (250)	55.4 (97)	31.0 (153)	<0.001
Anticoagulant therapy on admission, % (*n*)	11.1 (74)	15.8 (27)	9.5 (47)	0.025
BMI, kg/m^2^, median (IQR)	26.3 (23.3–31.1)	28.1 (25.5–33.2)	25.8 (23.0–29.5)	<0.001
NIHSS score on admission, median (IQR)	7 (5–11)	7 (5–11)	7 (4–11)	0.964
NIHSS score 24 h, median (IQR)	4 (1–8)	4 (2–8)	4 (1–8)	0.762
NIHSS score 72 h, median (IQR)	3 (1–7)	3 (1–7)	3 (1–7)	0.236
ASPECTS, median (IQR)	10 (9–10)	10 (9–10)	10 (9–10)	0.372
mCTA score, median (IQR)	5 (4–5)	5 (4–5)	5 (4–5)	0.112
Blood glucose, mmol/L, median (IQR)	6.6 (5.8–8.3)	8.7 (6.8–11.5)	6.4 (5.6–7.4)	<0.001
Blood glucose ≥ 7.8 mmol/L, % (*n*)	30.4 (201)	60.6 (103)	20.0 (98)	<0.001
Leucocyte, G/L, median (IQR)	8.2 (6.7–10.2)	8.3 (6.9–10.6)	8.2 (6.6–10.0)	0.279
C-reactive protein, mg/L, median (IQR)	3.2 (1.4–7.0)	3.2 (1.6–7.9)	3.2 (1.4–6.3)	0.721
Creatinine level, mg/dL, median (IQR)	83 (70–101)	91 (74–113)	81 (68–97)	<0.001
Onset-to-door time, min, median (IQR)	85 (58–128)	89 (59–144)	83 (58–122)	0.075
Door-to-needle time, min, median (IQR)	52 (36–71)	52 (36–74)	52 (36–70)	0.965
MT performed, % (*n*)	18.7 (130)	18.1 (33)	18.9 (97)	0.817
IVT performed, % (*n*)	88.9 (618)	89.6 (163)	88.7 (455)	0.749
sICH, % (*n*)	3.6 (25)	7.8 (14)	2.2 (11)	<0.001
90-day mRS ≤ 2, % (*n*)	62.1 (422)	48.9 (87)	66.7 (335)	<0.001
90-day mRS > 2, % (*n*)	37.9 (258)	51.1 (91)	33.3 (167)	<0.001
90-day mortality, % (*n*)	14.3 (97)	21.9 (39)	11.6 (58)	<0.001
Stroke etiology (TOAST classification)				
Large-artery atherosclerosis, % (*n*)	25.6 (178)	24.2 (44)	26.1 (134)	0.606
Cardioembolic, % (*n*)	23.5 (163)	26.9 (49)	22.2 (114)	0.198
Small-vessel occlusion, % (*n*)	14.8 (103)	22.5 (41)	12.1 (62)	<0.001
Other determined etiology, % (*n*)	1.6 (11)	0.5 (1)	1.9 (10)	0.194
Unknown etiology, % (*n*)	34.5 (240)	25.8 (47)	37.6 (193)	0.004

Abbreviations: IQR, interquartile range; DM, diabetes mellitus; TIA, transient ischemic attack; NIHSS, National Institutes of Health Stroke Scale; SBP, systolic blood pressure; DBP, diastolic blood pressure; BMI, body mass index; ASPECTS, Alberta Stroke Program Early CT Score; mCTA, multiphase CT-angiography; MT, mechanical thrombectomy; IVT, intravenous thrombolysis; sICH, symptomatic intracranial hemorrhage; mRS, modified Rankin scale.

**Table 2:** Association of diabetes mellitus and admission hyperglycemia with clinical outcomes in the overall population.

	Diabetes Mellitus				Admission Hyperglycemia			
	Non-Adjusted OR (95% CI)	*p*-Value	Adjusted * OR (95% CI)	*p*-Value	Non-Adjusted OR (95% CI)	*p*-Value	Adjusted * OR (95% CI)	*p*-Value
90-day favorable outcome (mRS 0–2)	0.48 (0.34–0.68)	<0.001	0.50 (0.32–0.76)	0.001	0.43 (0.30–0.60)	<0.001	0.48 (0.32–0.72)	<0.001
90-day poor outcome (mRS > 2)	2.10 (1.48–2.97)	<0.001	2.02 (1.31–3.11)	0.001	2.35 (1.67–3.31)	<0.001	2.09 (1.39–3.14)	<0.001
90-day mortality	2.15 (1.37–3.36)	0.001	2.45 (1.35–4.47)	0.003	2.63 (1.68–4.14)	<0.001	2.42 (1.37–4.28)	0.002
sICH	3.80 (1.69–8.52)	0.019	4.32 (1.54–12.09)	0.005	4.50 (1.88–10.80)	0.001	4.61 (1.58–13.39)	0.005

Abbreviations: OR, odds ratio; CI, confidence interval; mRS, modified Rankin scale; sICH, symptomatic intracranial hemorrhage. * Adjusted: age, gender, NIHSS on admission, coronary artery disease, C-reactive protein, intravenous thrombolysis, stroke onset-to-door time.

**Table 3:** Clinical characteristics of MT subgroup.

	Total Population (*n* = 130)	DM Present (*n* = 33)	DM Absent (*n* = 97)	*p*-Value
Age, years, mean (SD)	68 (13)	71 (10)	67 (13)	0.193
Gender, female, % (*n*)	53.1 (69)	63.6 (21)	49.5 (48)	0.159
Hypertension, % (*n*)	81.3 (104)	96.8 (30)	76.3 (74)	0.011
Hyperlipidemia, % (*n*)	59.2 (77)	63.6 (21)	57.7 (56)	0.713
Atrial fibrillation, % (*n*)	41.5 (51)	54.8 (17)	37.0 (34)	0.080
Coronary artery disease, % (*n*)	38.1 (40)	60.9 (14)	31.7 (26)	0.011
Previous stroke/TIA, % (*n*)	20.9 (24)	36.0 (9)	16.7 (15)	0.035
Antiplatelet therapy on admission, % (*n*)	30.6 (37)	41.4 (12)	27.2 (25)	0.148
Anticoagulant therapy on admission, % (*n*)	28.1 (34)	33.3 (10)	26.4 (24)	0.462
BMI, kg/m^2^, median (IQR)	26.1 (23.7–31.5)	26.0 (23.9–30.1)	31.1 (23.0–35.1)	0.289
NIHSS score on admission, mean (SD)	14 (7)	14 (8)	13 (7)	0.773
NIHSS score 24 h, mean (SD)	9 (8)	8 (9)	9 (8)	0.262
NIHSS score 72 h, mean (SD)	8 (7)	9 (8)	7 (7)	0.656
ASPECTS, median (IQR)	9 (8–10)	9 (8–10)	9 (8–10)	0.593
mCTA score, median (IQR)	4 (3–5)	4 (3–5)	4 (3–5)	0.568
Blood glucose, mmol/L, median (IQR)	7.0 (6.1–8.3)	7.9 (6.9–11.9)	6.6 (6.1–7.8)	0.001
Blood glucose ≥ 7.8 mmol/L, % (*n*)	33.3 (38)	59.3 (16)	25.3 (22)	0.001
Leucocyte, G/L, median (IQR)	9.1 (7.6–11.4)	9.3 (7.5–10.8)	9.0 (7.7–11.9)	0.692
C-reactive protein, mg/L (IQR)	3.6 (1.4–7.6)	3.5 (1.4–7.2)	4.2 (2.4–11.5)	0.402
Creatinine, mg/dL, median (IQR)	85 (72–107)	96 (73–119)	83 (72–100)	0.055
Occlusion location, % (*n*)				
ICA intracranial	20 (26)	15.2 (5)	21.6 (21)	0.420
MCA M1	46.9 (61)	48.5 (16)	46.4 (45)	0.835
MCA M2	22.3 (29)	27.3 (9)	20.6 (20)	0.428
Other	10.8 (14)	9.1 (3)	11.3 (11)	0.719
IVT prior MT, % (*n*)	40.8 (53)	21.2 (7)	47.4 (46)	0.008
Onset-to-door time, min, median (IQR)	79 (54–111)	84 (66–116)	75 (52–106)	0.201
Onset-to-GP time, min, median (IQR)	230 (182–300)	270 (213–310)	217 (175–292)	0.122
GP-to-revascularization time, min, median (IQR)	35 (20–60)	37 (22–62)	35 (19–57)	0.365
Onset-to-revascularization time, min, median (IQR)	273 (217–355)	305 (252–369)	257 (215–336)	0.192
Aspiration catheter, % (*n*)	86.2 (112)	84.8 (28)	86.6 (84)	0.802
Combined MT, % (*n*)	13.8 (18)	15.2 (5)	13.4 (13)	0.802
mTICI ≥ 2b, % (*n*)	87.6 (113)	81.8 (27)	89.6 (86)	0.243
Intraprocedural complications, % (*n*)	9.7 (11)	10.3 (3)	9.5 (8)	0.898
sICH, % (*n*)	9.2 (12)	18.2 (6)	6.2 (6)	0.040
90-day mRS ≤ 2, % (*n*)	38.4 (50)	24.2 (8)	43.3 (42)	0.046
90-day mRS > 2, % (*n*)	61.5 (80)	75.8 (25)	56.7 (55)	0.046
90-day mortality, % (*n*)	26.1 (34)	39.3 (13)	21.6 (21)	0.037

Abbreviations: IQR, interquartile range; DM, diabetes mellitus; TIA, transient ischemic attack; NIHSS, National Institutes of Health Stroke Scale; BMI, body mass index; ASPECTS, Alberta Stroke Program Early CT Score; mCTA, multiphase CT-angiography; ICA, internal carotid artery; MCA, middle cerebral artery; MT, mechanical thrombectomy; IVT, intravenous thrombolysis; GP, groin puncture; mTICI, modified thrombolysis in cerebral infarction; sICH, symptomatic intracranial hemorrhage; mRS, modified Rankin scale.

**Table 4:** Association of diabetes mellitus and admission hyperglycemia with clinical outcomes in the MT subgroup.

	Diabetes Mellitus				Admission Hyperglycemia			
	Non-Adjusted OR (95% CI)	*p*-Value	Adjusted * OR (95% CI)	*p*-Value	Non-Adjusted OR (95% CI)	*p*-Value	Adjusted * OR (95% CI)	*p*-Value
90-day favorable outcome (mRS 0–2)	0.40 (0.16–0.99)	0.050	0.62 (0.18–2.15)	0.449	0.13 (0.05–0.38)	<0.001	0.14 (0.04–0.51)	0.003
90-day poor outcome (mRS > 2)	2.51 (1.01–6.27)	0.050	2.32 (0.54–10.00)	0.259	7.72 (2.66–22.40)	<0.001	6.99 (1.98–24.72)	0.003
90-day mortality	2.52 (1.04–6.07)	0.040	3.72 (1.04–13.34)	0.044	3.39 (1.39–8.26)	0.007	3.76 (1.11–12.76)	0.034
sICH	3.37 (1.01–11.31)	0.049	12.45 (1.73–89.60)	0.012	4.07 (1.11–14.89)	0.034	7.36 (1.26–44.18)	0.029
Successful recanalization	0.52 (0.17–1.57)	0.249	0.39 (0.09–1.67)	0.205	0.61 (0.19–1.90)	0.392	0.42 (0.09–1.97)	0.274

Abbreviations: OR, odds ratio; CI, confidence interval; mRS, modified Rankin scale; sICH, symptomatic intracranial hemorrhage. * Adjusted: age, gender, NIHSS on admission, coronary artery disease, thrombolysis performed, stroke onset-to-door time, recanalization status.

## Data Availability

The data presented in this study are available on request from the corresponding author. The data are not publicly available due to patient privacy considerations (HIPPA).

## Supplementary Materials

The following supporting information can be downloaded at: https://www.mdpi.com/article/10.3390/life12050632/s1, Table S1: Clinical characteristics of the IVT subgroup; Table S2: Association of diabetes mellitus and admission hyperglycemia with clinical outcomes in the IVT subgroup.
