# Supplementary material for: The Impact of Diabetes Mellitus and Admission Hyperglycemia on Clinical Outcomes after Recanalization Therapies for Acute Ischemic Stroke: STAY ALIVE National Prospective Registry
Source: Life (Basel). 2022 Apr 25;12(5):632. doi: 10.3390/life12050632 (PMC9147213; doi:10.3390/life12050632)
Supplement: Supplementary file 1 [file life-12-00632-s001.zip › life-1666215-supplementary.pdf]

life

MDPI

Supplementary Materials

The Impact of Diabetes Mellitus and Admission Hyperglycemia on Clinical Outcomes after Recanalization Therapies for Acute Ischemic Stroke: STAY ALIVE National Prospective Registry

Peter Janos Kalmar, Gabor Tarkanyi, Zsofia Nozomi Karadi, Laszlo Szapary * and Edit Bosnyak

Department of Neurology, Medical School, University of Pecs, 7624 Pecs, Hungary; kalmar.peter@pte.hu (P.J.K.); tarkanyi.gabor@pte.hu (G.T.); karadi.zsofia@pte.hu (Z.N.K.); bosnyak.edit@pte.hu (E.B.)

* Correspondence: ptestroke@gmail.com; szapary.laszlo@pte.hu

Table S1. Clinical characteristics of IVT subgroup.

	Overall Population (*n* = 565)	DM Present (*n* = 149)	DM Absent (*n* = 416)	*p*–Value
Age, years, median (IQR)	69 (60–77)	72 (64–78)	67 (59–76)	<0.001
Genders, female, % (n)	43.2 (244)	43.0 (64)	43.3 (180)	0.947
Hypertension, % (n)	78.7 (444)	91.9 (136)	74.0 (308)	<0.001
Hyperlipidaemia, % (n)	62.5 (340)	69.9 (102)	59.8 (238)	0.032
Atrial fibrillation, % (n),	12.2 (68)	16.1 (23)	10.9 (45)	0.101

Coronary artery disease, % (n)	21.2 (117)	37.8 (54)	15.4 (63)	<0.001
Previous stroke/TIA, % (n)	22.2 (124)	28.0 (40)	20.2 (84)	0.053
Antiplatelet therapy on admission, % (n)	38.9 (213)	58.2 (85)	31.9 (128)	<0.001
Anticoagulant therapy on admission, % (n)	7.4 (40)	12.1 (17)	5.7 (23)	0.013
NIHSS score on admission, median (IQR)	6 (4–10)	7 (4–10)	6 (4–10)	0.783
BMI, kg/m^2, median (IQR)	26.4 (23.2–30.8)	27.5 (25.6–32.9)	25.7 (22.8–29.4)	<0.001
NIHSS score 24 hours, median (IQR)	3 (1–7)	4 (2–8)	3 (1–7)	0.226
NIHSS score 72 hours, median (IQR)	3 (1–7)	3 (1–7)	3 (1–7)	0.148
ASPECTS, median (IQR)	10 (9–10)	10 (9–10)	10 (9–10)	0.470
mCTA score, median (IQR)	5 (4–5)	5 (4–5)	5 (4–5)	0.630
Blood glucose, mmol/L, median (IQR)	6.6 (5.7–8.3)	8.8 (6.8–11.5)	6.4 (5.6–7.3)	<0.001
Admission hyperglycemia, % (n)	29.8 (163)	60.8 (87)	18.8 (76)	<0.001

Leucocyte, G/L, median (IQR)	8.1 (6.6–9.8)	8.3 (6.8–10.4)	7.9 (6.5–9.6)	0.142
C–reactive protein, mg/L, median (IQR)	3.1 (1.4–6.7)	2.9 (1.4–7.9)	3.1 (1.4–6.3)	0.947
Creatinine level, mg/dL, median (IQR)	83 (69–100)	90 (74–108)	80 (67–97)	<0.001
Onset–to–door time, min, median (IQR)	87 (59–130)	90 (58–148)	86 (60–127)	0.168
Door–to–needle time, min, median (IQR)	52 (36–72)	51 (35–73)	52 (36–71)	0.853
sICH, % (n)	2.3 (13)	5.4 (8)	1.2 (5)	0.004
90–day mRS ≤ 2, % (*n*)	65.8 (372)	53.0 (79)	70.4 (293)	<0.001
90–day mRS > 2, % (*n*)	34.2 (193)	47.0 (70)	29.6 (123)	<0.001
90–day mortality, % (*n*)	11.2 (63)	17.4 (26)	8.9 (37)	0.004

Abbreviations: IQR, interquartile range; DM, Diabetes mellitus; TIA, transient ischemic attack; NIHSS, National Institutes of Health Stroke Scale; SBP, systolic blood pressure; DBP, diastolic blood pressure; BMI, body mass index; ASPECTS, Alberta Stroke Program Early CT Score; mCTA, multiphase CT–angiography; MT, mechanical thrombectomy; IVT, intravenous thrombolysis; sICH, symptomatic intracranial hemorrhage; mRS, modified Rankin scale

Table S2. Association of diabetes mellitus and admission hyperglycemia with clinical outcomes in IVT subgroup

	Diabetes Mellitus				Admission Hyperglycemia			
	**Non–Adjusted OR (95% CI)**	***p* Value**	**Adjusted* OR (95% CI)**	***p* Value**	**Non–Adjusted OR (95% CI)**	***p* Value**	**Adjusted* OR (95% CI)**	***p* Value**
90–day favorable outcome (mRS 0–2)	0.47 (0.32–0.70)	<0.001	0.50 (0.32–0.79)	0.003	0.51 (0.35–0.74)	<0.001	0.58 (0.37–0.90)	0.014
90–day poor outcome (mRS > 2)	2.11 (1.43–3.10)	<0.001	1.99 (1.27–3.14)	0.003	1.97 (1.35–2.88)	<0.001	1.73 (1.12–2.69)	0.014
90–day mortality	2.17 (1.26–3.72)	0.005	2.15 (1.10–4.19)	0.025	2.46 (1.43–4.25)	0.001	2.00 (1.05–3.80)	0.035
Hemorrhagic transformation	4.65 (1.50–14.45)	0.008	4.27 (1.22–14.91)	0.023	4.83 (1.43–16.28)	0.011	4.88 (1.24–19.18)	0.023

Abbreviations: OR, odds ratio; CI, confidence interval; mRS, modified Rankin Scale; sICH, symptomatic intracranial hemorrhage; *Adjusted: age, gender, NIHSS on admission, coronary artery disease, C–reactive protein, intravenous thrombolysis, stroke onset–to–door time.
